# Comparing the efficacy of intravenous morphine versus ibuprofen or the combination of ibuprofen and acetaminophen in patients with closed limb fractures: a randomized clinical trial

**DOI:** 10.1186/s12873-024-00933-y

**Published:** 2024-01-25

**Authors:** Mehdi Nasr Isfahani, Hossein Etesami, Omid Ahmadi, Babak Masoumi

**Affiliations:** 1 Department of Emergency Medicine, School of Medicine, Isfahan University of Medical Sciences, Isfahan, Iran; 2 Student Research Committee, Vice Chancellery for Research, Isfahan University of Medical Sciences, Isfahan, Iran; 3 Trauma Data Registration Center, Al-Zahra University Hospital, Isfahan University of Medical Sciences, Isfahan, Iran

**Keywords:** Pain, Fracture, Limb, Morphine, Ibuprofen, Acetaminophen

## Abstract

**Introduction:**

This study aims to investigate the effectiveness of intravenous ibuprofen or intravenous ibuprofen plus acetaminophen compared to intravenous morphine in patients with closed extremity fractures.

**Methods:**

A triple-blinded randomized clinical trial was conducted at a tertiary trauma center in Iran. Adult patients between 15 and 60 years old with closed, isolated limb fractures and a pain intensity of at least 6/10 on the visual analog scale (VAS) were eligible. Patients with specific conditions or contraindications were not included. Participants were randomly assigned to receive intravenous ibuprofen, intravenous ibuprofen plus acetaminophen, or intravenous morphine. Pain scores were assessed using the visual analog scale at baseline and 5, 15, 30, and 60 min after drug administration. The primary outcome measure was the pain score reduction after one hour.

**Results:**

Out of 388 trauma patients screened, 158 were included in the analysis. There were no significant differences in age or sex distribution among the three groups. The pain scores decreased significantly in all groups after 5 min, with the morphine group showing the lowest pain score at 15 min. The maximum effect of ibuprofen was observed after 30 min, while the ibuprofen-acetaminophen combination maintained its effect after 60 min. One hour after injection, pain score reduction in the ibuprofen-acetaminophen group was significantly more than in the other two groups, and pain score reduction in the ibuprofen group was significantly more than in the morphine group.

**Conclusion:**

The study findings suggest that ibuprofen and its combination with acetaminophen have similar or better analgesic effects compared to morphine in patients with closed extremity fractures. Although morphine initially provided the greatest pain relief, its effect diminished over time. In contrast, ibuprofen and the ibuprofen-acetaminophen combination maintained their analgesic effects for a longer duration. The combination therapy demonstrated the most sustained pain reduction. The study highlights the potential of non-opioid analgesics in fracture pain management and emphasizes the importance of initiation of these medications as first line analgesic for patients with fractures. These findings support the growing trend of exploring non-opioid analgesics in pain management.

**Trial registration:**

ClinicalTrials.gov Identifier: NCT05630222 (Tue, Nov 29, 2022). The manuscript adheres to CONSORT guidelines.

## Introduction

Bone fractures are a common injury among patients presenting with trauma. Each year, approximately 2.5% of people in Iran suffer from new fractures, which is similar to the global fracture rate [1]. Additionally, about 50% of people experience at least one fracture during their lifetime [2]. Of these, almost 90% are extremity fractures, and most are closed fractures [3]. Fractures can cause intense acute pain, and physicians should consider appropriate pain control for their patients [4].

Acute pain is the most common symptom in patients with bone fractures and results from the stimulation of pain receptors due to tissue damage. The pain interferes with the treatment process and should be relieved before clinical assessment if it is severe. Despite the importance of pain control in the emergency department, patients’ pain severity is usually underdiagnosed, and half of the patients are dissatisfied with the quality of their pain management [5–7]. Therefore, to manage pain in patients with bone fractures, some interventions should be considered, such as immobilizing and elevating the affected area and using an ice pack on the site. Although these interventions can decrease pain intensity, medications are usually required for adequate pain relief [8].

Opioids are the traditional group of analgesics used for pain relief, with known efficacy. Morphine, the most well-known opioid, is considered the gold standard drug for analgesia in fracture pain, and other analgesics are compared with it for their efficacy [9, 10]. Although morphine is an effective pain reliever, its side effects make physicians hesitant to prescribe it. For instance, morphine administration not only leads to general side effects such as sedation and respiratory suppression, but it can also increase non-union fractures and delay healing in bone fractures [11–13]. As a result, analgesics from other types of drugs can be considered as substitute drugs [14, 15].

Recently, NSAIDs have been considered the primary choice over opioids due to their suitable analgesic effect and fewer side effects. Ibuprofen, one of the most commonly prescribed NSAIDs, is indicated in single or combined form as an alternative to opioids because of its lower side effects and similar analgesic effect. For example, two studies have reported that ibuprofen does not significantly differ from morphine in decreasing the pain intensity of fracture pain in children [16, 17]. Another study in patients with arm fractures indicated that ibuprofen is as effective as acetaminophen-codeine [18]. In the meantime, there is no strong evidence that short-term use of NSAIDs for analgesia after fracture is deleterious to healing. And there is limited evidence to suggest that prostaglandins promote bone formation and that NSAIDs might inhibit the process. This issue has not been thoroughly pursued or established through properly conducted studies [19]. Additionally, some studies suggest a combination of drugs to replace opioids or decrease the dose. Acetaminophen is a drug that can be administered alone or in combination with other drugs, such as NSAIDs [20]. In combination therapy, not only the dosage of each drug is decreased, but physicians also have the opportunity to suppress pain with more than one mechanism [21, 22].

Despite the occurrence of side effects of opioids in both adults and children, researchers have focused on replacing opioids with other types of analgesics in pediatric medicine for fracture pain [8, 16, 18]. Furthermore, previous studies have usually focused on oral analgesics prescribed as outpatient or after discharge. However, in this study, we aim to investigate the effect of intravenous ibuprofen or intravenous ibuprofen plus acetaminophen compared to intravenous morphine in patients with closed extremity fractures.

## Materials and methods

### Design and setting

We conducted a triple-blinded randomized clinical trial study (clinicaltrials.gov Identifier: NCT05630222) from autumn 2022 to winter 2022 at Kashani Hospital (a tertiary trauma center), Isfahan, Iran.

### Participants

Any adult patient with trauma referring to the emergency department whose physical examination suggested limb fracture was eligible for this study. Our inclusion criteria included adults between 15 and 60 years old with closed, isolated limb fracture (confirmed by radiographs and physical examination) whose pain intensity was at least 6/10 on the visual analog scale (VAS). Additionally, patients were not included if they had any of these involvements: any respiratory or hemodynamic or neurologic problems due to trauma, analgesic use within 6 h prior to ED arrival, routine analgesic use because of chronic pain, renal or hepatic failure, allergy to the study drugs, history of gastrointestinal bleeding, lung disease such as asthma, head trauma during trauma, drug abuse, unable to explain pain according to the VAS score, pregnancy, concurrent neurovascular damage, multiple fractures, or other pain hiding fracture pain. Patients were excluded if they were unwilling to continue the survey, or undergoing emergency surgery. Drug adverse events were documented during one hour of intervention.

### Intervention

Primary care including ice compression, limb immobilization and elevation was done for all patients. Every patient received drugs in the same appearance and volume in this method: The ibuprofen group received 800 mg ibuprofen in 100 ml of normal saline for 15 min and 5 ml of normal saline for 5 min, intravenously; The ibuprofen-acetaminophen group received 400 mg of ibuprofen plus 1 gr of acetaminophen in 100 ml of normal saline for 15 min and 5 ml of normal saline for 5 min, intravenously. The morphine group received 100 ml of normal saline for 15 min and 0.1 mg/kg of morphine sulfate, increasing volume to 5 ml with normal saline for 5 min, intravenously. Previous studies guided the selection of drug doses [3, 15, 20, 23, 24].

### Outcome

After fixing the fractured limb, elevation, and ice compression, the patient’s pain score was assessed via VAS score because of its validity and easiness to use [25, 26]. VAS score is a self-reported pain rating score which is a scaled line beginning from 0 cm (no pain) to 10 cm (worst pain). Patients mark the line for reporting their pain used to comparing pain intensity between different times or patients [27]. We assessed the pain severity of the patients at baseline and 5, 15, 30, and 60 min after the beginning of the infusion of drugs via the VAS scale. Our primary outcome was assessing the reduction in pain score of the groups after one hour from injection.

### Sample size calculation

Sample size was calculated based on minimum clinically significant mean difference of 1.3 point between groups and standard deviation of 2.3 points for the VAS. Our null hypothesis was that there would be no statistically significant difference of VAS between groups after one hour from injection. The power of this study and alpha value were considered 80% and 0.05 respectively. Finally, we calculated the sample size to be 50 patients for each group. To decrease the margin of error we added 10% to the sample size, that is to say the expected sample size was estimated to be 55 for each arm.

### Randomization and blinding

All trauma patients transported to the ED via EMS or personal vehicle, who were assigned triage level of 2 or 3 according to the emergency severity index (ESI) triage system, were considered eligible for the study. Patients were divided into three groups by a random number table produced by the RANDOM.ORG website. As per the randomization table, the emergency medicine specialist provided three drug groups with the same appearance and volume. A general practitioner (GP) visited patients to record information and pain scores. Besides, another medical staff, a nurse, administered the drugs according to the codes. The general practitioner and nurse did not know about each other and were not included in the data analysis done by statistician. Therefore, the patients, the general practitioner, the nurse and the statistician were blinded about intervention groups. A nurse with clinical responsibilities opened a pre-coded envelope with details of the drug and randomization number. The drugs dosage was chosen based on the previous studies. This nurse was not involved in the administration of analgesia, the assessment of the patient, or the treatment of adverse effects. The principal researcher unlocked the code of any patient who showed signs of moderate to severe drug reactions or adverse effects, and the patient was excluded from the study. Moderate to severe drug reaction or adverse effect is implying to anaphylactic shock, angioedema, respiratory failure, neurologic complications or gastrointestinal bleeding. The research GP or the nurse, with non-clinical duties, and the patient were blinded to the treatment. Notably, the project involved a team comprising of GPs, nurses, medical students, and emergency medicine specialists, who worked on rotational basis, so as to cover a 24/7 recruitment program. Furthermore, in a face-to-face training class, the former three groups acquired or boosted their practical skills including history taking, physical exams, and administering medication.

### Statistical analysis

We used the Kolmogorov-Smirnov test to evaluate normality of numerical data. Data were presented as mean ± standard deviation (mean ± SD) if normally distributed and median ± interquartile range (median ± IQR) if not, or n (%). Independent sample T, paired sample T, one way ANOVA, and Chi-square tests were also used for evaluation of the hypothesis. An intention to treat approach was considered for analysis and a significance level of less than 0.05 was considered in all analyses. Finally, the collected data were entered into BMI SPSS software (ver. 22).

## Results

**Fig. 1:**
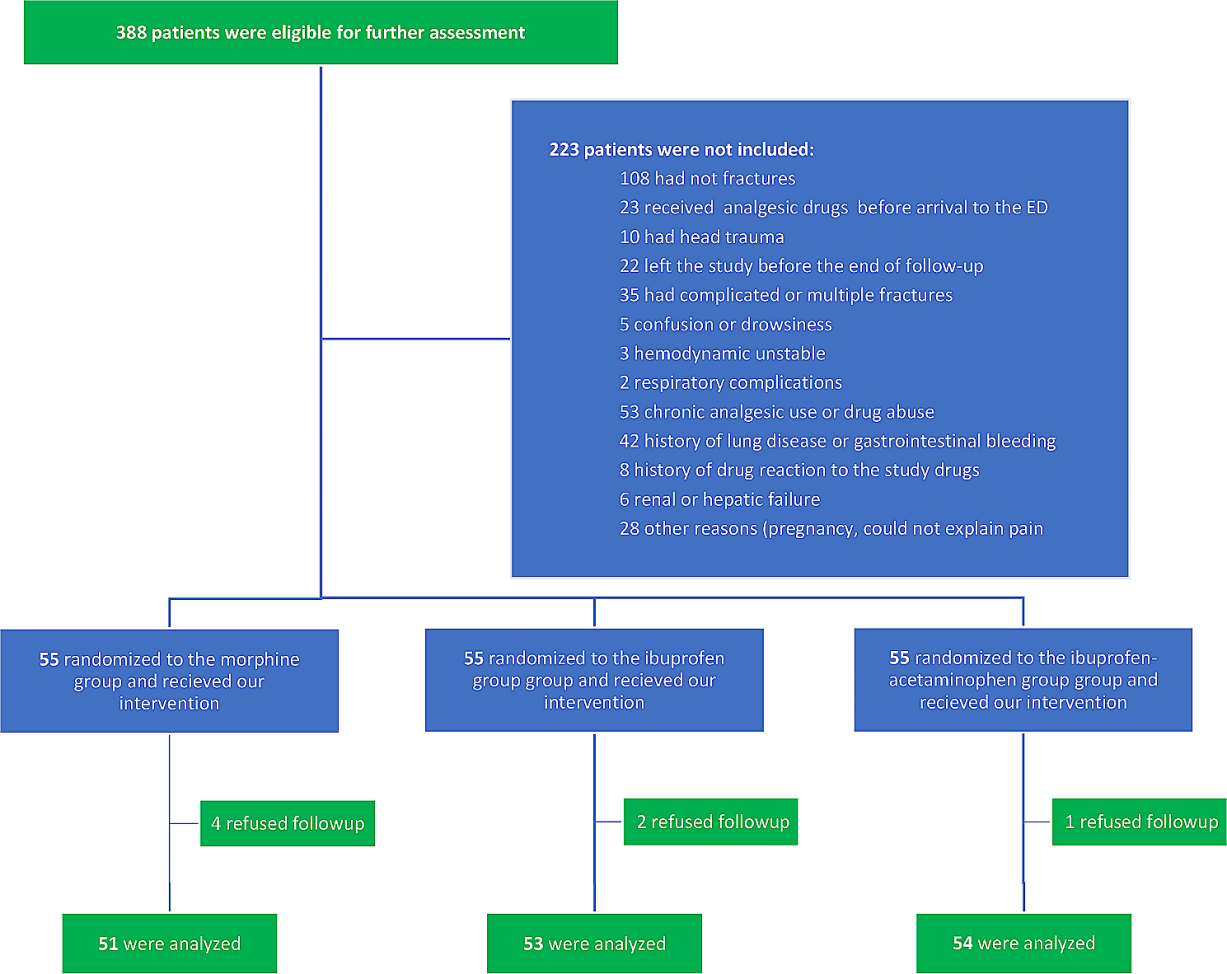
The consort flowchart

Out of 388 trauma patients who were clinically suspicious, 165 were included in the study after radiography and complete evaluation. Ultimately, 7 patients were excluded and 158 patients were analyzed (Fig. 1). The patients were predominantly male (78%) with a mean age of 34 ± 12, and the three groups did not differ significantly with respect to age (*p*-value: 0.115) or sex (*p*-value: 0.725) (Table 1). Mild complications such as pain or irritation at the site of injection did not differ between the three groups of study and none of the patients had moderate to severe drug reaction during one hour follow up (*p*-value: 0.321) (Table 1). In terms of fracture type, about 42 percents of the patients had upper extremity fractures rather than lower extremity fractures (*p*-value: 0.221) (Table 1). Furthermore, most of the patients were presented to the emergency department with ambulance transportation (*p*-value: 0.207) (Table 1).

**Table 1:** Characteristics of patients

	All	Morphine	Ibuprofen	Ibuprofen-Acetaminophen	*p*-value
No. of patients	158	51	53	54	-
Female sex, No. (%)	35 (22)	12 (24)	13 (25)	10 (19)	0.725
Age, mean (SD)	34 (12)	31 (11)	35 (12)	35 (12)	0.115
Baseline pain score, mean (SD)	7.9 (1.3)	7.7 (1.26)	8.2 (1.4)	7.9 (1.2)	0.176
Minor side effects, No. (%)	24 (15)	5 (10)	8 (15)	11 (20)	0.321
Upper extremity fractures, No. (%)	67 (42)	17 (33)	23 (43)	27 (50)	0.221
Ambulance transportation, No. (%)	128 (81)	40 (78)	47 (89)	41 (76)	0.207

Before treatment, the pain scores for the morphine, ibuprofen, and ibuprofen-acetaminophen groups were 7.7 ± 1.26, 8.2 ± 1.4, and 7.9 ± 1.2, respectively, and the three groups were not significantly different in terms of pain score (*p*-value: 0.176) (Table 1).

Five minutes after injection, pain scores decreased significantly in all groups with the mean ± SD of 4.6 ± 1.6 for the morphine group (*p*-value < 0.01), 7.3 ± 1.4 for the ibuprofen group (*p*-value < 0.01), and 7.8 ± 1.3 for the ibuprofen-acetaminophen group (*p*-value: 0.01). At this time, the pain score in the morphine group was significantly lower than in the other two groups (*p*-value < 0.01).

**Fig. 2:**
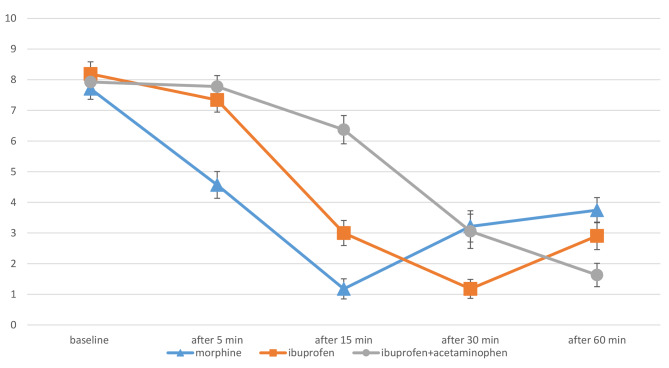
The pain score in the three groups during one hour

Fifteen minutes after injection, the mean ± SD pain scores were 1.2 ± 1.2, 3.0 ± 1.6, and 6.4 ± 1.8 for the morphine, ibuprofen, and ibuprofen-acetaminophen groups, respectively. At the 15-minute mark, the pain score in the morphine group was significantly lower than in the ibuprofen group, and the pain score in the ibuprofen group was significantly lower than in the ibuprofen-acetaminophen group (*p*-value < 0.01) (Fig. 2).

Thirty minutes post-injection, the pain score in the morphine group increased with the mean ± SD of 3.2 ± 1.9 (*p*-value < 0.01), while the scores in the ibuprofen and ibuprofen-acetaminophen groups continued to decrease with the mean ± SD of 1.2 ± 1.1 (*p*-value < 0.01) and 3.1 ± 2.1 (*p*-value < 0.01), respectively. At this point, the pain score in the ibuprofen group was significantly lower than in the other two groups (*p*-value < 0.01), while there was no significant difference between the morphine and ibuprofen-acetaminophen groups (*p*-value: 0.68) (Fig. 2).

Finally, one hour after injection, the pain scores in the morphine and ibuprofen groups increased with the mean ± SD of 3.8 ± 1.5 and 2.9 ± 1.7, respectively (*p*-value < 0.01), while the score in the ibuprofen-acetaminophen group continued to decrease with the mean ± SD of 1.6 ± 1.4 (*p*-value < 0.01). At this time, the pain level in the ibuprofen-acetaminophen group was significantly lower than in the ibuprofen group (*p*-value < 0.01), and the pain level in the ibuprofen group was significantly lower than in the morphine group (*p*-value < 0.01).

In Table 2, the focus is on the reduction in pain scores rather than the baseline pain levels. The results indicate that one hour after injection, the acetaminophen-ibuprofen group exhibited a significantly greater reduction in pain scores compared to the ibuprofen group (*p*-value < 0.01). Additionally, the ibuprofen group showed a greater reduction in pain scores compared to the morphine group (*p*-value < 0.01). The table also reveals the time intervals at which the most significant reduction in pain scores occurred: 15 min after injection in the morphine group, 30 min after injection in the ibuprofen group, and one hour after injection in the acetaminophen-ibuprofen group (Table 2).

**Table 2:** Decline in pain scores during the study

Decline in pain, mean (CI)	Morphine (1)	Ibuprofen (2)	Ibuprofen-Acetaminophen (3)	*p*-value (1,2)	*p*-value (1,3)	*p*-value (2,3)
Decline in pain after 5 min	3.1 (2.7 to 3.5)	0.9 (0.6 to 1.2)	0.1 (0 to 0.2)	**< 0.01**	**< 0.01**	< 0.01
Decline in pain after 15 min	6.5 (6.1 to 6.9)	5.2 (4.8 to 5.6)	1.6 (1.3 to 1.9)	**< 0.01**	**< 0.01**	< 0.01
Decline in pain after 30 min	4.5 (3.8 to 5.2)	7.0 (6.6 to 7.4)	4.9 (4.4 to 5.4)	**< 0.01**	**0.366**	< 0.01
Decline in pain after 1 h	4.0 (3.4 to 4.6)	5.3 (4.8 to 5.8)	6.3 (5.9 to 6.7)	**< 0.01**	**< 0.01**	< 0.01

No moderate to severe complications were reported during the 1-hour follow-up period after injection (Table 1).

## Discussion

In our study, we found pain level in all groups decreased early in minute 5. However, patients who received morphine experienced their lowest pain level after 15 min of intervention and then the analgesic effect of morphine decreased. The maximum effect of ibuprofen was observed after 30 min and interestingly the effect of ibuprofen-acetaminophen combination maintained after 60 min. Therefore, the findings show that, the effect of ibuprofen and its combination with acetaminophen initiate as early as morphine and moreover can affect for a longer period of time. However, considering minimum clinically important difference, we will get in another conclusion about initiation of effects.

We know there were statistically significant changes in pain level between all the time points in each group, but some of these changes were not clinically important. In previous studies, a difference of 1.3 in pain levels between two time points has been considered clinically significant [23]. Using this criterion, we observed a plateau in the morphine group regarding mean pain level after 30 min from injection, while in the other two groups, the analgesia effect initiates 15 min post-injection and all the changes after that are significant rather than previous time. Although this difference is clinically significant, pain intensity is typically categorized into three levels in pain management guidelines, and pain scores under 4 points are categorized as mild pain that can be managed with non-pharmacologic treatment or a low-dose oral analgesic, if requested by the patient [28, 29]. So, while the mean VAS score increased in the ibuprofen and morphine groups, it did not require invasive intervention, because their pain score consistently after 15 min from injection was categorized as mild pain. The pain score in the ibuprofen-acetaminophen group was consistently categorized as mild pain from 30 min after injection until the end of the study.

In recent years, there has been a growing trend towards replacing opioids with non-opioid drugs, and various studies have compared the efficacy of these drugs in trauma patients. While these studies have used different drugs, doses, and surveyed patients at different time periods and age categories, they have focused on the effect of non-opioids, especially ibuprofen and acetaminophen, versus opioids.

Furthermore, most of these studies have been conducted in the field of pediatric medicine [23, 30–32]. In one of these studies, the combination of ibuprofen and acetaminophen was found to be as effective as acetaminophen with oxycodone, hydrocodone, or codeine, which are three types of opioids, in patients with acute extremity pain after two hours of treatment. In this study, the drugs were administered orally, and the doses were 400 mg of ibuprofen and 1000 mg of acetaminophen, 5 mg of oxycodone and 325 mg of acetaminophen, 5 mg of hydrocodone and 300 mg of acetaminophen, or 30 mg of codeine and 300 mg of acetaminophen [18], Other studies have also demonstrated that ibuprofen or acetaminophen, or their combinations, are as effective as opioids [31, 32]. These studies have focused on patients with limb pain or extremity trauma; however, it should be noted that the pain experienced by patients with a fractured limb can be significantly greater than other types of pain [33].

Morphine is considered the gold standard opioid for the management of severe pain, but it has several side effects in fracture physiology [14] and is a short-acting analgesic in fracture pain management as we saw in this study [34]. In contrast, ibuprofen and acetaminophen have longer periods of action than morphine, which is consistent with our result [35, 36]. Additionally, several studies have shown that the combination of acetaminophen with ibuprofen has a synergistic effect that is greater than the prescription of each drug alone in post-operative patients, which may be due to different analgesic pathways [37–39].

The national opioid crisis is a pressing issue concerning public health policies, as increasing opioid abuse has become a great burden for countries worldwide [40]. Prescribing opioids for patients with pain chief complaint not only increases the risk of opioid abuse, but also has side effects from one-time consumption [41]. While our data only discusses morphine alternatives in the first hour of admission, it can prompt physicians to consider continuing treatment with non-opioids during admission or after discharge. Moreover, it can help increase society’s acceptance of non-opioids as effective even in emergency departments, ultimately protecting patients from their first encounter with opioids and potentially contributing to the fight against the opioid crisis.

### Limitations

Our study had some limitations. First, we followed patients for one hour after injection of the drugs. Although further follow-up could provide more data about pain and side effects, our study was limited to one hour after drug injection as we aimed to determine whether a non-opioid drug can relieve pain during the patients’ stay in the emergency department, where most patients are referred to orthopedic services or discharged quickly, making it challenging to follow them and consider rescue analgesic drugs.

Second, our study only focused on moderate to severe side effects due to the limited one-hour follow-up period, which is a relatively short time frame to carefully assess the incidence of mild or long-term side effects [10, 14, 25, 26, 34].

Third, there are various forms of opioid and non-opioid drugs, and while this study focused on the parenteral form of three drugs, other studies and pain management guidelines recommend different drugs and doses, indicating the need for further research [25, 26].

Fourth, the target population in this study consisted of patients with simple extremity fractures. While our findings may apply to other types of traumas, it is possible that different types of fractures could result in different pain patterns. In addition, it is important to assess pain with different origins, such as post-surgical pain or visceral pain, separately from the findings of this study.

Finally, some proportions of patients were presented to the ED by personal vehicles, rather than ambulance. Of course, by using emergency severity index (ESI) triage system to meticulously sort trauma patients, based on the severity of injury and resource needed, it seems the risk of bias has been decreased.

## Conclusion

In conclusion, our study compared non-opioid parenteral drugs with morphine in the ED during the first hour of admission when patients experience the most pain. Our findings indicate that ibuprofen and its combination with acetaminophen were more effective than morphine one hour after injection, and provided a longer period of analgesic effect. Further studies are needed to assess the long-term safety and efficacy of these non-opioid alternatives, as well as the feasibility of their implementation in EDs. Nonetheless, our results provide valuable insights for improving pain management strategies and reducing the use of opioid medications in the acute care setting.

## Data Availability

The datasets generated and analyzed during the current study are not publicly available due to privacy, but are available from the corresponding author on reasonable request.
